# Modeling the Kinetics of Potassium Diffusion in Estima Potato under Different Leaching Conditions

**DOI:** 10.1155/2020/1876463

**Published:** 2020-11-24

**Authors:** David Adu-Poku, Selina A. Saah, Jacob K. Agbenorhevi

**Affiliations:** ^1^Department of Chemical Sciences, University of Energy and Natural Resources, Sunyani, Ghana; ^2^Department of Food Science and Technology, Kwame Nkrumah University of Science and Technology, Kumasi, Ghana

## Abstract

The diffusion of potassium in potato (*Solanum tuberosum*) at different leaching conditions was investigated. Two modes of pretreatment of potato samples (cubes and spheres) by preheating to 80°C and leaching at temperatures of 20-80°C were performed using a temperature- and agitation-controlled batch extractor. A Fickian model incorporating the effective diffusion coefficient (*D*_eff_), partition coefficient (*K*) between the solute concentration in the potato and medium, and mass transfer coefficient (kl) was developed to simulate and predict the fraction of potassium leached from the potato at any temperature. Results showed significant reduction in activation energies from 92 to 25.02 kJ/mol for cubes and from 75.02 to 13.40 kJ/mol for spheres culminating in higher extraction rates when samples were preheated to 80°C. The *D*_eff_, *K*, and kl values obtained were in the range of 0.02 − 7.33 × 10^−9^  m^2^/s, 0.63 − 8.00 × 10^−2^, and 0.01333.00 × 10^−4^  m/s, respectively. The kinetic parameters showed a change in slope or discontinuity in the gelatinization temperature range as a function of temperature, an indication of a change in the diffusional matrix. The optimum operating conditions were 80°C preheating and leaching at temperatures up to 50°C. The proposed mathematical model offered a satisfactory description of both dynamic and equilibrium mass transfers of potassium by adequately predicting the fraction of potassium from potato cubes and spheres. The present findings could be useful in the pretreatment of potato for renal patients.

## 1. Introduction

A critical step in the preparation of potato for renal patients is leaching, a process intended to extract potassium from the potato [1]. Potato offers one of the most affordable sources of potassium with the potassium level per fresh weight considerably higher than banana, orange, mushroom, milk, avocado, raisin, orange, salmon, spinach, and tomato [2]. Potato supplies 18% of the total potassium intake of Brits, followed by meat and meat products (15%), dairy products (13%), cereals and cereal products (13%), and vegetables (10%) [3]. Although potassium is important to the human body, people with compromised kidney have to completely avoid or severely restrict their intake of potassium-rich foodstuffs, such as potato (>250 mg/100 g), in order to limit their daily intake to within a 1.7 mg to 2.5 mg range [4].

Despite the promising health benefits, renal patients are restricted to only 150 g of boiled potatoes or 75 g of homemade chips per day. Even then, the potato must be cut up and soaked prior to cooking to allow potassium to leach. However, the effectiveness of this recommended procedure in removing potassium has been widely questioned [1]. Earlier research by Bethke and Jansky (2008) reported that leaching alone no matter the duration is ineffective in removing potassium from potato. Other studies, however, show that other processes such as shredding, which has a tendency of increasing the surface area before leaching, can decrease the potassium content up to 17% [5]. Other techniques such as normal cooking (boiling) and double cooking (boil, rinse, and boil again) have also reported reductions of 34% and 67%, respectively [6]. Nonetheless, a comprehensive study that enables kinetic parameters to be determined for effective pretreatment of potato for renal patients is still lacking.

The ease of potassium removal in potato is significantly hindered by structural barriers offered by the cellular tissues of the tuber. In our earlier study, we demonstrated that there were significant variations in the cellular architecture, moisture content, and distribution, which influence the diffusion of potassium through and out of potatoes [7]. It was further established that by preheating samples to 50 and 80°C, the amount of potassium leached at each stage of sampling for leaching at 30°C increased by five- (5-) and eight- (8-) folds, respectively. Solid-liquid extraction principles have often been employed to extract bioactive compounds from natural sources, and therefore, various mass transfer-based mechanistic mathematical models are available in the literature [8–10]. Mass transfer during a solid-liquid extraction represents a solute diffusion in a media contacting a well-stirred solution of finite volume. Whilst empirical models have often been used to describe the concentration kinetic of extractable solutes in extract during solid-liquid extraction [11, 12], the extractable solute effective diffusivity (*D*_eff_) has frequently been estimated by different methods characterized by concentration at a punctual given time [10, 13]. By building concentration-time profiles of mass transfer processes and quantifying the diffusion coefficients, researchers have characterized diffusion processes in carrots [14], green peas and kiwi fruits [15], and cereals (oats and grains) and green beans [16], as a way of assessing the effect of critical variables.

Prominent methods for kinetic modelling and effective diffusivity estimations include regression fit of the analytical solutions of diffusion equation considering the infinite volume of solvent [17, 18], direct nonlinear regression of differential equations [19, 20], or an equation valid at very short times (*τ* < 0.0189) [21]. These different points of view of solid-liquid extraction often result in different values even under similar processing conditions [22]. It will therefore be ideal to develop a unified model that is deduced from the fundamental principles of mass transfer and thermodynamics and capable of predicting the mass transfer phenomenon at different conditions than those used to estimate the mass transfer properties. Food materials, like potato, are complex in nature as they have heterogeneous, amorphous, hygroscopic, and porous properties [23]. Changes in the microstructure of food material during processing significantly affect solute mass transfer. Whilst some studies have reported that diffusion through a solid matrix is impossible and that diffusion only occurs in the liquid contained in the matrix or *via* the gas phase [22], others contend that diffusion through cellular walls and membranes does actually occur [24, 25]. Potato tissues act as a physiological barrier to mass and heat transfers, but by heating above 50°C, the cell walls and membranes acting as an ultrafiltration membrane break down. It was demonstrated that significant microstructural changes including complete gelatinization of starch, damaged outlines of cell walls and cell membranes, and the cellular cements in the matrix do occur at 80°C thereby allowing water, sugars, and salts to diffuse freely.

The objective of this study, therefore, was to determine the kinetic parameters necessary to optimize the extraction of potassium from potato and propose a mathematical model for the prediction of potassium extraction as a function of time.

## 2. Materials and Methods

### 2.1. Materials

An oval-shaped, medium sized (6-8 cm diameter), and light yellow-skinned Estima potato (*Solanum tuberosum*) was obtained from a local Sainsbury supermarket (UK). The potatoes, grown in Norfolk and Shropshire and available all year-round in the UK, were stored in the laboratory at room temperature and analysed within five days after purchase. Tubers were washed under running water, wiped with blotting paper, and hand peeled with a stainless-steel vegetable peeler. With a kitchen knife and a stainless-steel melon baller, potato cubes (2.0 cm side length) and spheres (1.4 cm radius) were made from the peeled tuber. Dimensions were checked using a Vernier calliper correct to 0.025 cm. The moisture content of samples was thermogravimetrically determined using the oven dry method [26].

### 2.2. Sample Pretreatment

Preheating affects the cell structure of potato tissues and the degree of microstructural deformation and integrity changes positively with temperature. The mode of preheating was informed by our previous study which established the gelatinization temperature range and confirmed complete gelatinization by 80°C [7]. Two modes of sample preheating were adopted: preheating to 80°C and also to temperatures of leaching. Prior to leaching, the samples were preheated to temperatures of interest between 20 and 80°C in an oven.

Temperature monitoring was performed with a thermocouple to within ±0.2°C.

### 2.3. Kinetic Leaching Experiments

The solid-liquid extraction operation was performed according Hasanzadeh and Souraki (2016) at different temperatures (20, 25, 30, 35, 40, 45, 50, 55, 60, 65, 70, 75, and 80°C). Deionized water and natural convectional operation were used throughout the experiments. The product-to-solution mass ratio was 9 : 500 for cubes and 3 : 100 for spheres.

**Figure 1:**
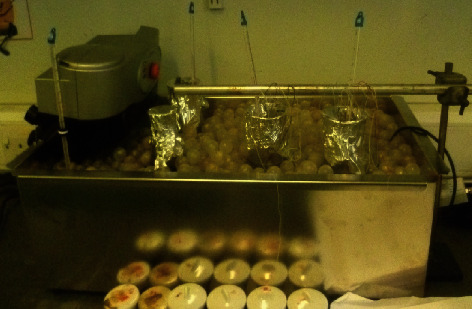
Experimental set-up for isothermal leaching of potato samples.

In each isothermal experiment, a covered 1000 mL beaker containing 500 mL deionized water and a 2 L reservoir were placed in a thermostatically and magnetically stirred water bath (2mag magnetic motion, Munich, Germany) set at 1200 rpm as in Figure 1. To prevent evaporation and loss of heat, the water bath was covered with floating bath-insulating balls before heating. The temperature control was maintained within ±0.2°C.

When the temperature of the water in the beaker and the bath was at the required leaching temperature, a preweighed sample was lowered into the beaker and the timer started. Isothermal leaching was allowed at a constant water volume of 500 mL for 4 h (as the duration was generally sufficient for the attainment of equilibrium at the 20–80°C range). At each sampling time (5, 10, 15, 20, 25, 30, 40, 60, 90, 120, 150, 180, 210, and 240 min), 10 mL samples were taken, and after each sample had been removed, 10 mL of deionized water at the same temperature from the reservoir was added to maintain a constant volume. The leached samples and the sampled liquid were all analysed for the content of potassium using Flame Atomic Absorption Spectroscopy (AAnalyst 100, Perkin Elmer, USA). For potassium analysis, five standard solutions of KCl in the concentration range of 0.05 to 2.00 (mg K/L) were prepared whilst deionized water was used as a blank. Similarly, standard solutions (0.04 to 0.5 mg/L) of NaCl and MgCl_2_ were, respectively, used as Na and Mg standards for the determination of their contents. All measurements were carried out in triplicates and reported as averages.

### 2.4. Modelling the Kinetics of Mass Transfer, Partition, and Diffusion Coefficients

By neglecting the internal and external heat transfer effects, the initial thermal transient effect, any shrinkage, and the external movement resistance, simple analytical solutions of Fick's second law of diffusion have been developed for slab and spherical geometries using appropriate initial and boundary conditions [22, 27, 28]. These analytical solutions have helped simplify the determination of effective diffusivity and other mass transfer properties in complex food systems. In this study, a simulation model drawn from various mathematical relations connecting the essential kinetic and thermodynamic parameters capable of characterizing potassium mass transfer during leaching of potato spheres and cubes under specific assumptions was developed.

Under conditions of surface evaporation, the simplest practical assumption is that the rate of exchange of mass is directly proportional to the difference between the actual concentration in the surface at any time and that needed to maintain equilibrium with the surrounding water. With the assumption that a sphere is initially at a uniform concentration and there is a surface condition, the solution of the classical Fickian diffusion equation for the total amount of diffusing substance entering or leaving the sphere which links the mass of diffusant (*M*) with time (*t*) under surface evaporation as given in Cranks [29] is

(1) $$\begin{array}{c} \frac{M_{t}}{M_{\infty }}=1-\overset{\infty }{\underset{n=1}{\sum }}\frac{6L^{2}\exp\left(-\beta_{n}^{2}D_{\text{eff}}t/a^{2}\right)}{\beta_{n}^{2}\left\{\beta_{n}^{2}+L\left(L-1\right)\right\}}, \end{array}$$

where *β*_*n*_s are the roots of

(2) $$\begin{array}{c} \beta_{n}\cot\beta_{n}+L-1=0. \end{array}$$

Rearranging equation (1) gives

(3) $$\begin{array}{c} \frac{M_{t}}{M_{\infty }}=\overset{\infty }{\underset{n=1}{\sum }}\frac{6L^{2}\exp-\left(\beta_{n}^{2}D_{\text{eff}}t/a^{2}\right)}{\beta_{n}^{2}\left\{\beta_{n}^{2}+L\left(L-1\right)\right\}}. \end{array}$$

A key assumption made under the surface evaporation approach for cubic samples was that the potato cubes behave in a similar way as the spherical samples during the diffusion process. This was necessary as potatoes for cooking are often cut in cubes or slabs. To facilitate the application of equation (3) to cubic samples, the amount of deviation in the boundary measurement between potato cubes and spheres was calibrated by a correction factor. The volumetric mass transfer coefficient (kla), which is the product of mass transfer coefficient (kl) and the interfacial area of sample per unit volume of water (*a*), and effective diffusion coefficient (*D*_eff_) are related to the time lag, *L*, by

(4) $$\begin{array}{c} L=\frac{\text{kla}}{D_{\text{eff}}}. \end{array}$$

The partition factor, *K*, between the solute in equilibrium in the sphere and the solution is also related to the amount of solute at infinite time, *M*_∞_, by

(5) $$\begin{array}{c} M_{\infty }=\frac{1}{1+V_{p}/KV_{w}}, \end{array}$$

where *V*_*w*_ is the volume of water and *V*_*p*_ is the boundary volume of potato samples.

Due to the difficulty in having to rearrange equation (3) into the form of a straight line, simulation runs aimed at getting the best fit with the experimental data were adopted for this study.

Making *L* the subject of equation (2),

(6) $$\begin{array}{c} L=1-\beta \cot\beta , \end{array}$$

Given cot*β* = 1/tan*β*,

(7) $$\begin{array}{c} L=1-\frac{\beta }{\tan\beta }. \end{array}$$

From a table of *L* and *β* using equation (7), a simulation curve was constructed. Relevant input parameters including the effective diffusion coefficient (*D*_eff_), partition coefficient (*K*) between the solute concentration in potato and medium, overall mass transfer coefficient for potassium diffusion into water (kl), equilibrium concentration of solute (*M*_∞_), surface area of sample (Sap), volume of water (*V*_*w*_), boundary volume of sample (*V*_*s*_), and half thickness of sample (*l*(*a*)) were incorporated into the simulation model as a way of determining kinetic parameters for the diffusion process. Estimates of *D*_eff_, *K*, and kl were made for the different treatment conditions.

Using the first six roots (*β*1–*β*6) of equation (3), the fraction of potassium leached from the potatoes was predicted.

### 2.5. Activation Energy

The activation energy is another important kinetic parameter that represents the energy barrier that separates two minima of potential energy (of the reactants and products of a reaction) which has to be overcome by reactants to commence a chemical reaction [30]. Kinetic and thermodynamic parameters of mass transfer processes are usually determined from the kinetic extraction curves at a range of precise temperatures. The relationship between effective diffusivity and temperature (*T*) often follows a first-order rate process described by the Arrhenius equation:

(8) $$\begin{array}{c} D_{\text{eff}}=D_{0}\exp\left(-\frac{E_{a}}{RT}\right), \end{array}$$

where *D*_0_ is the preexponential factor of the Arrhenius equation (m^2^/s), *E*_*a*_ is the activation energy for the potassium diffusion (kJ/mol), *R* is the ideal gas constant (J/mol K), and *T* (K) is the leaching temperature. The activation energy was calculated by plotting ln(*D*_eff_) vs. the reciprocal of the temperature.

### 2.6. Statistical Evaluation of Kinetic Data

Data analysis was done using Origin 2018 (OriginLab Corporation, Northampton, Massachusetts). The reported results in this work are the averages of at least three measurements. Analysis of variance (ANOVA, *α* = 0.05) was employed to test the significance of differences between three or more treatment groups whilst an independent sample *T*-test (*α* = 0.05) was used to compare two treatment groups.

## 3. Results and Discussion

### 3.1. Distribution of Magnesium, Potassium, and Sodium in the Fresh Potato

**Figure 2:**
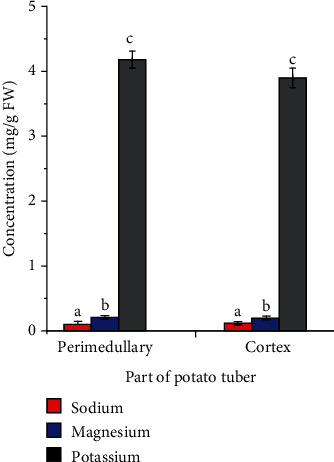
Distribution of potassium, sodium, and magnesium in the perimedullary and cortex parts of potato tuber. ^a–c^Bars of the same mineral (same colour) with the same alphabets are not significantly different (*p* > 0.05).

In assessing the mineral distribution within the tuber and its potential effect on diffusion of such minerals through and out of the tuber, levels of potassium, sodium, and magnesium in samples from the cortex (region between the skin and the vascular ring) and perimedullary (the region between the pith and the vascular ring) regions of the tuber were analysed. The results revealed potassium concentrations of 3.90 ± 0.15 mg/g FW and 4.18 ± 0.13 mg/g FW, sodium concentrations of 0.12 ± 0.03 mg/g FW and 0.10 ± 0.02 mg/g FW, and magnesium concentrations of 0.20 ± 0.11 mg/g FW and 0.20 ± 0.02 mg/g FW in the cortex and perimedullary regions, respectively. With the exception of potassium where the level in the perimedullary (though not significantly different; *p* > 0.05) was higher, the levels of sodium and magnesium appear evenly distributed between the cortex and the perimedullary (Figure 2).

Although some studies on the distribution of minerals in potato reported that the magnesium and potassium were concentrated at the centre whilst the sodium level decreased towards the centre [31], the results from this study were not statistically adequate enough for such a conclusion. Given the even distribution of potassium between the two regions of the tuber, samples for kinetic studies were taken from any part of the tuber.

### 3.2. Potassium Kinetic Leaching Curves

**Figure 3:**
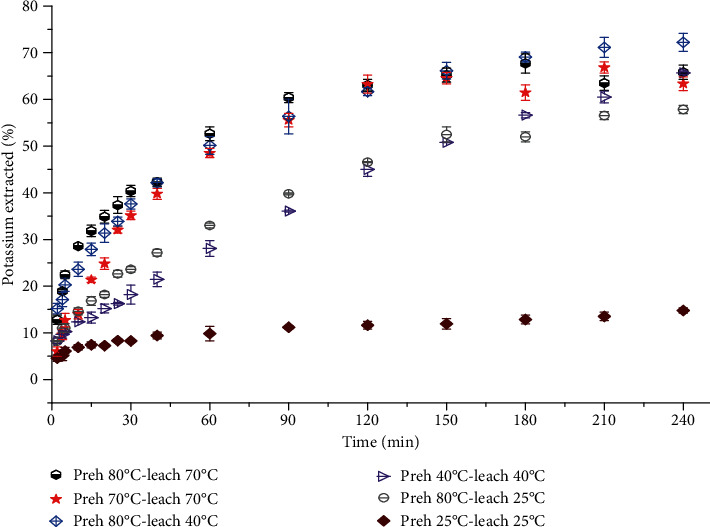
Leaching curves for potato spheres of different pretreatments leached at 25, 40, and 70°C for 4h.

Preheating, as a pretreatment technique generally, increased the rate of potassium diffusion out of the potato attributable to the breakdown of cellular walls and membranes. Increasing temperature of leaching water also increased the rate of extraction. The variation in the rate of potassium leaching was highly significant within the first hour of leaching. Whilst increased rates were significant (*p* = 0.05) for leaching at pregelatinization temperatures (<50°C), the effect of preheating at, say 80°C prior to leaching, appeared not to meaningfully affect the extraction rate at or above 50°C leaching. For instance, by preheating samples to 80°C, the amount of potassium extracted at each stage of sampling for leaching at the pregelatinization temperatures either doubled or quadrupled. The leaching curves typically followed similar trends as in shown in Figure 3.

This therefore suggests that preheating potato samples at temperatures above 50°C and leaching below the same would make significant impact on the level of potassium in potato for kidney-compromised people.

The observed diffusional trend is also consistent with the assumption that at the start of the leaching process, the rate of potassium diffusion from the surface of the potatoes into the water was quite high, but as the leaching proceeded, and this potassium on the surface was removed, the rate of diffusion of the potassium through the potato to the surface became rate limiting. Equilibrium time was reached within 4 hrs of leaching at the various temperatures (except 20 and 25°C) for the different treatments and shapes.

### 3.3. Estimation of Mass Transfer, Partition, and Effective Diffusion Coefficients of Potassium

**Figure 4:**
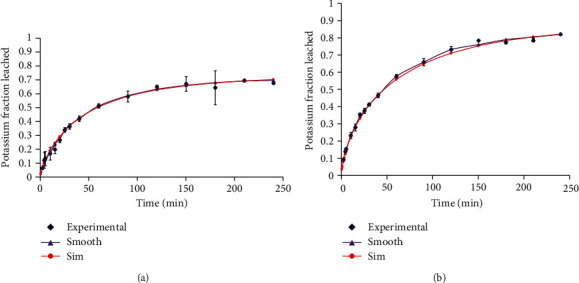
Sample model simulation for the diffusion of potassium in potato (a) spheres preheated and leached at 70°C and (b) cubes preheated at 80°C and leached at 70°C (error bars: standard deviation).

The mass transfer coefficient (kl), partition coefficient (*K*), and effective diffusion coefficient (*D*_eff_) of potassium were estimated from the simulation model built from equations (4), (5), and (7). Smoothing of the experimental data was done by adjusting, in line, data points considered to have deviated much from the general trend. The simulated curve was then adjusted to optimally fit the data trend. Estimates of kl, *K*, and *D*_eff_ were made for each sample at specific temperatures. Typical examples of the model simulation under the different pretreatments are shown in Figure 4.

**Figure 5:**
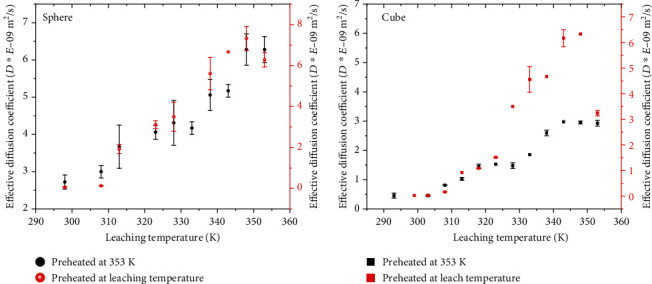
Effect of temperature on effective diffusion coefficients (*D*_eff_) of potassium in preheated potatoes.

The *D*_eff_ values for the cubes ranged from 0.02 × 10^−9^ m^2^/s to 6.33 × 10^−9^ m^2^/s whilst those of the spheres ranged from 0.06 × 10^−9^ m^2^/s to 7.33 × 10^−9^ m^2^/s over the 20–80°C temperature range. Generally, the effective diffusion coefficients increased linearly over the pregelatinization temperatures (<55°C) and inconsistently over the gelatinization temperatures (55–75°C) and showed a declining rate over the postgelatinization temperatures (>75°C) as in Figure 5.

These observed *D*_eff_ values were consistent with published literature such as (0.2–10) × 10^−9^m^2^/s in potato, carrot, onion, and green pepper at 60–80°C [32, 33] and 10^−12^–10^−8^m^2^/s in peas at 60–80°C [34]. These potassium diffusivity values were slightly lower than those of water and NaCl diffusivities ((8.2 − 12.3) × 10^−9^m^2^/s) obtained during osmotic dehydration of potato at 25-55°C [35] but compare favourably with those of KCl (1.82 × 10^−9^m^2^/s) at 25°C [36]. The 0.02 × 10^−9^ to 7.33 × 10^−9^m^2^/s range obtained in this study is also within the diffusional rates cited for liquids (10^−8^ to 10^−9^m^2^s^−1^) and solids (10^−11^ m^2^s^−1^) [22]. This indicates that the diffusion of potassium through the potato over the 20–80°C temperature range was predominantly through the liquid medium. Significant variations in *D*_eff_ between samples preheated at 80°C and those at the temperature of leaching over narrow ranges of temperature were also observed. The linear rate of diffusion at the subgelatinization temperatures is consistent with the Stokes-Einstein equation in that it was inversely proportional to the viscosity of the water as the potato matrix consisted mainly of intact ungelatinized starch granules of varying sizes [7]. As the temperature of the potato increased, solubilization of pectic substances along with swelling resulting from the absorption of water by the starch granules is reported to occur [37]. As a process, gelatinization imposes different microstructural changes on the potato starch which in turn affect the diffusion dynamics of potassium in potatoes. The breaks in the *D*_eff_ within the gelatinization temperature range reflected changes in diffusional matrix of potassium through and out of the tissue. The reduced rate of diffusion at the postgelatinization temperatures was partly attributed to the increased viscosity resulting from cell gel formation composed of starch granules embedded in an amylose matrix. The path for potassium diffusion within this temperature range was through a gel of higher viscosity. With many diffusional paths now blocked, drag on the molecules increased due to the smaller walls of the diffusional path. It is reported that gelatinized starch in excess water reassociates into an ordered structure in order to retrieve a crystal order [37–39], and this may have partly hindered the rate of potassium diffusion.

**Figure 6:**
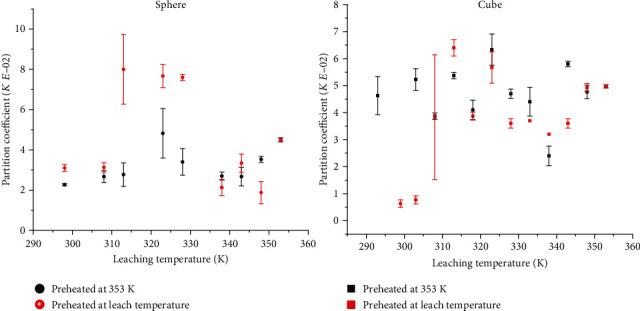
Effect of temperature on partition coefficient of potassium in preheated potatoes.

**Figure 7:**
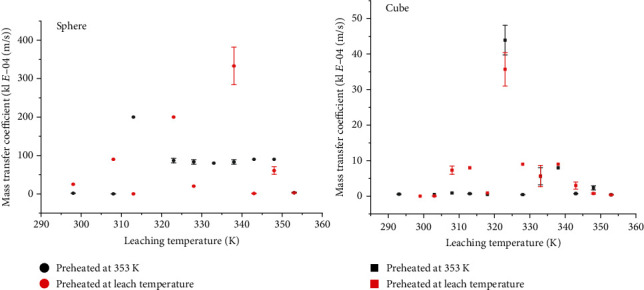
Effect of temperature on mass transfer coefficient of potassium in preheated potatoes.

Estimates of the partition and mass transfer coefficients ranged from (0.63–8.00) × 10^−2^ to (0.01–333.00) × 10^−4^m/s, respectively. The general trends in the partition and mass transfer coefficients with temperature were similar to that of the effective diffusion coefficients, increased linearly over the pregelatinization temperatures (<328 K) and inconsistently over the gelatinization temperatures (328–348 K), and showed a declining rate over the postgelatinization temperatures (>348 K) (Figures 6 and 7). The huge deviations observed in the gelatinization temperature region could be linked with inconsistent changes in the microstructure during the gelatinization of potato starch.

The mass transfer and partition coefficients did not change significantly, an indication that the potassium diffusion out of the potato is less dependent on mass transfer and partition coefficients under the tested conditions. This could partly be linked with the negligible external mass transfer resistance under the experimental conditions created by infinite water volume and high mass ratio between the solution and the product.

An analysis of variance (ANOVA) conducted to assess the degree of variation between the different pretreatments also showed significant interaction between the leaching temperature and the pretreatment. For instance, for the spherical samples, a statistically significant main effect was observed for the leaching temperature (*F*_0.05,8,9_ = 6.22, *p* < 0.01) with larger size effect (eta‐squared = 0.847) compared with the lesser size effect (eta‐squared = 0.025) for the pretreatment technique.

### 3.4. Activation Energy of Potassium Diffusion

**Figure 8:**
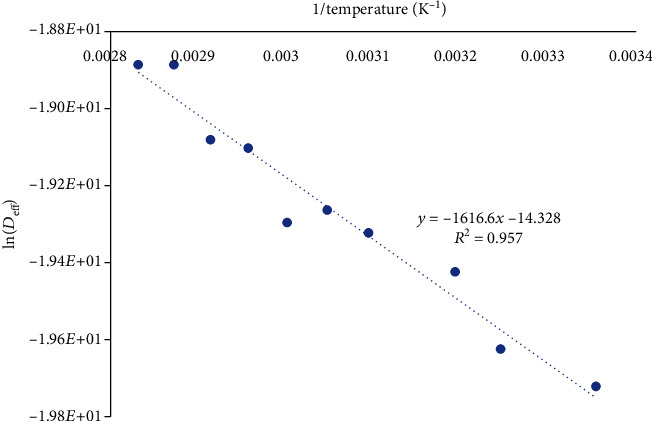
Arrhenius plot for the diffusion of potassium in potato spheres preheated at 80°C.

The activation energy of the diffusion process was calculated by plotting the natural logarithm of *D*_eff_ against the reciprocal of the leaching temperature (Figure 8).

The plots were found to be linear within the range of temperatures studied for the different treatments, indicating Arrhenius dependence. The temperature dependence of the effective diffusivity for the different pretreatments and shapes was represented as follows:

- Cubes preheated at the leach temperature:

(9) $$\begin{array}{c} D_{\text{eff}}=6.38\times 10^{5}\exp\left(\frac{-92003.37}{RT}\right) \end{array}$$

(ii) Cubes preheated at 80°C:

(10) $$\begin{array}{c} D_{\text{eff}}=1.97\times 10^{-5}\exp\left(\frac{-25109.45}{RT}\right) \end{array}$$

(iii) Spheres preheated at leach temperature:

(11) $$\begin{array}{c} D_{\text{eff}}=1.77\times 10^{3}\exp\left(\frac{-75016.12}{RT}\right) \end{array}$$

(iv) Cubes preheated at 80°C:

(12) $$\begin{array}{c} D_{\text{eff}}=5.99\times 10^{-7}\exp\left(\frac{-13440.47}{RT}\right) \end{array}$$

The observed activation energy values ranged from 13.44 to 92 kJ/mol over the temperature range of interest. By preheating the potato samples to 80°C, significant reductions in the energy barriers were detected. For cubes, 66.89 kJ/mol reduction was observed whilst the spheres saw 61.58 kJ/mol reduction. Though the activation energies of potatoes preheated at leach temperature were relatively higher due to higher physiological resistance (sizable 3-dimensional cubes and spheres as opposed to the usual 2-dimesional slab many adopt), the range of activation energy values was not only reflective of the dynamics of the kinetic parameters over the 20–80°C range but was also consistent with published literature values such as 17-43 kJ/mol for water diffusion in starchy materials [40] and 50.1 kJ/mol for ascorbic acid in peas kJ/mol [41].

### 3.5. Model Prediction of Kinetic Parameters

**Figure 9:**
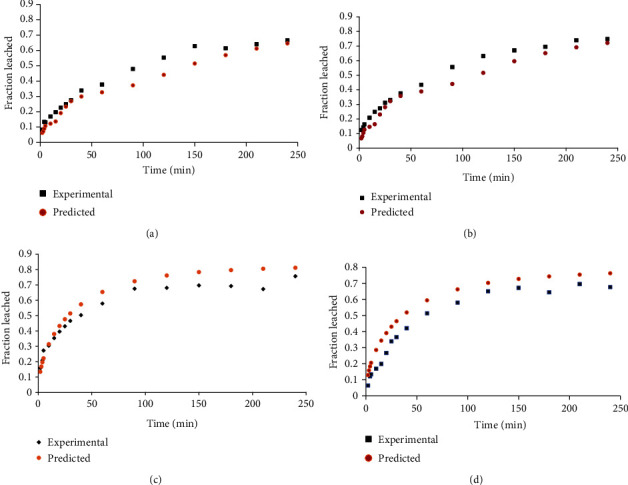
Experimental and predicted fraction of potassium leached from potato (a) cubes preheated at 80°C and leached at 30°C, (b) cubes preheated at 80°C and leached at 50°C, (c) cubes preheated and leached at 65°C, and (d) spheres preheated and leached at 70°C.

In validating the predictive performance of the model, the first six roots (*β*1-*β*6) of equation (3) were used to predict the fraction of potassium leached from the potatoes under the two pretreatment techniques over the 20–80°C temperature range. For the 80°C preheated samples and those which were preheated above 40°C, the independent sample *t*-test revealed no statistically significant variations between the predicted and observed fractions of leached potassium at 95% confidence level (*p* > 0.05) as reflected in Figure 9.

**Figure 10:**
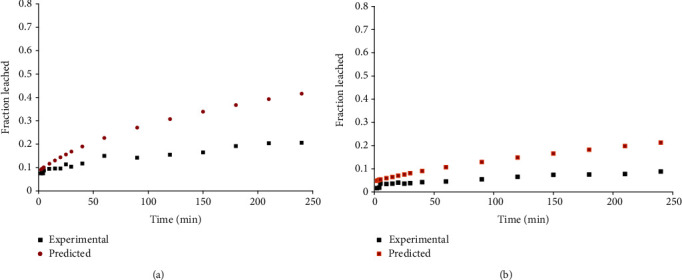
Experimental and predicted fraction of potassium leached from potato (a) spheres preheated and leached at 35°C and (b) cubes preheated and leached at 30°C.

The model, however, overpredicted the fraction of potassium leached below 40°C largely due to its inability to account for the higher physiological resistance offered by the cellular tissues as in Figure 10.

## 4. Conclusion

In this study, kinetic parameters governing the extraction of potassium from potato in water were investigated. By preheating samples to 80°C, the activation energies were reduced from 92 to 25.02 kJ/mol for cubes and from 75.02 to 13.40 kJ/mol for spheres. The optimum operating conditions were 80°C preheating and leaching at temperatures up to 50°C. Estimates of effective diffusion (0.02 − 7.33 × 10^−9^ m^2^/s), partition (0.63 − 8.00 × 10^−2^), and mass transfer (0.01333.00 × 10^−4^ m/s) coefficients were obtained by simulation of a proposed Fickian mathematical model. The proposed model offered satisfactory description of both dynamic and equilibrium mass transfers. The kinetic parameters showed a change in slope or discontinuity in and around the gelatinization temperature range as a function of temperature, an indication of a change in diffusional matrix. The proposed model adequately predicted the fraction of potassium from potato with good agreement between the predicted and the experimental, a significant step towards the determination of kinetic parameters for the enhanced removal of potassium from potato for renal patients.

## Data Availability

The experimental data used to support the findings of this study are available from the corresponding author upon request.

## References

[1] Cupisti A., Kovesdy C., D’Alessandro C., Kalantar-Zadeh K. (2018). Dietary approach to recurrent or chronic hyperkalaemia in patients with decreased kidney function. *Nutrients*.

[2] Braschi A., Naismith D. J. (2008). The effect of a dietary supplement of potassium chloride or potassium citrate on blood pressure in predominantly normotensive volunteers. *The British Journal of Nutrition*.

[3] Riley H. (2010). *Potato consumption in the UK–why is ‘meat and two veg’ no longer the traditional British meal?*.

[4] Hunsicker L. G., Modification of Diet in Renal Disease Study Group, Adler S. (1997). Predictors of the progression of renal disease in the modification of diet in renal disease study. *Kidney International*.

[5] Bethke P. C., Jansky S. H. (2008). The effects of boiling and leaching on the content of potassium and other minerals in potatoes. *Journal of Food Science*.

[6] Burrowes J. D., Ramer N. J. (2006). Removal of potassium from tuberous root vegetables by leaching. *Journal of Renal Nutrition*.

[7] Adu-poku D., Agbenorhevi J. K. (2017). Kinetics of potassium diffusion as influenced by microstructure of potato. *Am. J. Food Sci. Technol.*.

[8] Castillo-Santos K., Ruiz-López I. I., Rodríguez-Jimenes G. C., Carrillo-Ahumada J., García-Alvarado M. A. (2017). Analysis of mass transfer equations during solid-liquid extraction and its application for vanilla extraction kinetics modeling. *Journal of Food Engineering*.

[9] Silva L. P. S., Martínez J. (2014). Mathematical modeling of mass transfer in supercritical fluid extraction of oleoresin from red pepper. *Journal of Food Engineering*.

[10] Chen D., Zheng Y., Zhu X. (2012). Determination of effective moisture diffusivity and drying kinetics for poplar sawdust by thermogravimetric analysis under isothermal condition. *Bioresource Technology*.

[11] Chilev C., Koleva V., Simeonov E. (2014). A new empirical model for calculation the effective diffusion coefficient for solid–liquid extraction from plants. *Industrial and Engineering Chemistry Research*.

[12] Simeonov E., Yaneva Z., Chilev C. (2018). Kinetics of green solid-liquid extraction of useful compounds from plant materials: kinetics coefficients and modeling. *Green Processing and Synthesis*.

[13] Wang Y., Herdegen V., Repke J.-U. (2016). Identification and analysis of mass transfer coefficients and effective diffusion coefficients for models of solvent extraction of Montan wax. *Separation Science and Technology*.

[14] Andersson A., Gekas V., Lind I., Oliveira F., Öste R., Aguilfra J. M. (1994). Effect of preheating on potato texture. *Critical Reviews in Food Science and Nutrition*.

[15] Simal S., Femenia A., Garau M. C., Rosselló C. (2005). Use of exponential, Page’s and diffusional models to simulate the drying kinetics of kiwi fruit. *Journal of Food Engineering*.

[16] Walker L., Senadeera W. (2014). A variable diffusivity model for the drying of spherical food particulates. *Applied Mechanics and Materials*.

[17] Pedreschi F., Travisany X., Reyes C., Troncoso E., Pedreschi R. (2009). Kinetics of extraction of reducing sugar during blanching of potato slices. *Journal of Food Engineering*.

[18] Franco D., Sineiro J., Pinelo M., Núñez M. J. (2007). Ethanolic extraction of Rosa rubiginosa soluble substances: oil solubility equilibria and kinetic studies. *Journal of Food Engineering*.

[19] Nicolin D. J., Rossoni D. F., Jorge L. M. M. (2016). Study of uncertainty in the fitting of diffusivity of Fick’s second law of diffusion with the use of bootstrap method. *Journal of Food Engineering*.

[20] Garcia-Perez J. V., García-Alvarado M. A., Carcel J. A., Mulet A. (2010). Extraction kinetics modeling of antioxidants from grape stalk (Vitis vinifera var. Bobal): influence of drying conditions. *Journal of Food Engineering*.

[21] Cacace J. E., Mazza G. (2003). Mass transfer process during extraction of phenolic compounds from milled berries. *Journal of Food Engineering*.

[22] Varzakas T. H., Leach G. C., Israilides C. J., Arapoglou D. (2005). Theoretical and experimental approaches towards the determination of solute effective diffusivities in foods. *Enzyme and Microbial Technology*.

[23] Joardder M. U. H., Kumar C., Karim M. A. (2016). Food structure: its formation and relationships with other properties. *Critical Reviews in Food Science and Nutrition*.

[24] Aguilera J. M. (2005). Why food microstructure?. *Journal of Food Engineering*.

[25] Hasanzadeh R., Souraki B. A. (2016). Experimental and theoretical investigation of mass transfer during leaching of starch and protein from potato. *Chemical Engineering Communications*.

[26] da Silva Carneiro J., Nogueira R. M., Martins M. A., de Souza Valladão D. M., Pires E. M. (2018). The oven-drying method for determination of water content in Brazil nut. *Bioscience Journal*.

[27] Welti-Chanes J., Velez-Ruiz J. F. (2016). *Transport Phenomena in Food Processing*.

[28] Lazarides H. N., Gekas V., Mavroudis N. (1997). Apparent mass diffusivities in fruit and vegetable tissues undergoing osmotic processing. *Journal of Food Engineering*.

[29] Crank J. (1979). *The Mathematics of Diffusion*.

[30] Shafique Z., Mustafa M., Mushtaq A. (2016). Boundary layer flow of Maxwell fluid in rotating frame with binary chemical reaction and activation energy. *Results Phys.*.

[31] LeRiche E. L., Wang-Pruski G., Zheljazkov V. D. (2009). Distribution of elements in potato (Solanum tuberosum L.) tubers and their relationship to after-cooking darkening. *HortScience*.

[32] Kiranoudis C. T., Maroulis Z. B., Marinos-Kouris D., Saravacos G. D. (1994). Estimation of the effective moisture diffusivity from drying data. Application to some vegetables. *Developments in Food Engineering*.

[33] Zogzas N. P., Maroulis Z. B. (1996). Effective moisture diffusivity estimation from drying data. A comparison between various methods of analysis. *Drying Technology*.

[34] Zogzas N. P., Maroulis Z. B., Marinos-Kouris D. (1996). Moisture diffusivity data compilation in foodstuffs. *Drying Technology*.

[35] Pacheco-Angulo H., Herman-Lara E., García-Alvarado M. A., Ruiz-López I. I. (2016). Mass transfer modeling in osmotic dehydration: equilibrium characteristics and process dynamics under variable solution concentration and convective boundary. *Food and Bioproducts Processing*.

[36] Ribeiro A. C. F., Lobo V. M. M., Leaist D. G. (2005). Binary diffusion coefficients for aqueous solutions of lactic acid. *Journal of Solution Chemistry*.

[37] Bordoloi A., Kaur L., Singh J. (2012). Parenchyma cell microstructure and textural characteristics of raw and cooked potatoes. *Food Chemistry*.

[38] Singh J., Kaur L., Moughan P. J. (2012). Importance of chemistry, technology and nutrition in potato processing. *Food Chemistry*.

[39] Kaur A., Singh N., Ezekiel R., Guraya H. S. (2007). Physicochemical, thermal and pasting properties of starches separated from different potato cultivars grown at different locations. *Food Chemistry*.

[40] Panagiotou N. M., Krokida M. K., Maroulis Z. B., Saravacos G. D. (2004). Moisture diffusivity: literature data compilation for foodstuffs. *International Journal of Food Properties*.

[41] Singh N., Singh J., Kaur L., Sodhi N. S., Gill B. S. (2003). Morphological, thermal and rheological properties of starches from different botanical sources. *Food Chemistry*.

